# The Impact of Germination on Sorghum Nutraceutical Properties

**DOI:** 10.3390/foods9091218

**Published:** 2020-09-02

**Authors:** Nafiou Arouna, Morena Gabriele, Laura Pucci

**Affiliations:** National Research Council, Institute of Agricultural Biology and Biotechnology (IBBA), Research Unit of Pisa, Via Moruzzi 1, 56124 Pisa, Italy; nafiou.arounaa@gmail.com (N.A.); laura.pucci@ibba.cnr.it (L.P.)

**Keywords:** sorghum, germination, total phenolic content, antioxidant activity, CAA-RBC, bioactive peptides, angiotensin-converting enzyme (ACE) inhibitor activity

## Abstract

Sorghum is a gluten-free cereal representing a staple food in many countries of Africa, where germination is traditionally used for the preparation of several sorghum-based products. This study focused on the effect of germination on total phenolic content, in vitro and ex vivo antioxidant activity, and antihypertensive action of sorghum from Togo. Total phenolic content was estimated as Folin–Ciocalteu reducing capacity, while antioxidant activities were assessed using 2,2-diphenyl-1-picrylhydrazyl (DPPH) and Ferric Reducing Antioxidant Power (FRAP) in vitro tests and ex vivo by the cellular antioxidant activity (CAA) assay on human erythrocytes. The antihypertensive effect of germinated and non-germinated sorghum peptides fraction was evaluated as angiotensin-converting enzyme (ACE) inhibitory activity. Despite our findings demonstrated no impact of germination on the total phenolic content, non-germinated sorghum showed significantly higher in vitro antioxidant activities than the germinated one; further, non-germinated sorghum displayed significantly higher ACE inhibition than germinated sorghum that, instead, at lower doses, exhibited better erythrocytes protection from peroxyl radicals. In conclusion, the germination process negatively impacted the in vitro antioxidant activity and the antihypertensive effect of sorghum while improved erythrocytes protection. This study evidenced better nutraceutical potential of non-germinated sorghum that, besides good antioxidant activity, represents an important source of ACE-inhibitory peptides. However, the germination process might have positively impacted the profile of bioactive compounds involved in the protection of human erythrocytes from oxidative damage.

## 1. Introduction

Sorghum (*Sorghum bicolor*) is one of the most important cereal crops in the world after maize, wheat, barley, and rice, with a production of over 59 million tons in 2018, of which Africa produced more than 50% [1]. Because of its adaptability to arid conditions and extreme temperatures, sorghum is widely cultivated in semi-arid and arid areas, where it is mainly used as staple food [2]. Sorghum has been for centuries an important food source for populations in developing countries; nevertheless, there has been little interest in sorghum as a component of the diet in developed areas, such as Europe, Australia, and the United States, where it has been principally used as animal feeding and biofuel production [3,4]. Recently, there has been increased interest in using sorghum grains as a component of the human diet in developed areas, not only because it is a gluten-free cereal but also due to its high nutritional value and health-promoting potential [5].

The nutritional value and chemical composition of sorghum grains are similar to maize, rice, and wheat. Its main components are polysaccharides, followed by proteins and lipids. However, among cereals, sorghum has the least starch and protein digestibility because of the high bound of tannins to sorghum starch and proteins. Since starch is the principal source of calories from cereal grains and in human feeding, in general, starch binding is particularly relevant because it helps lower caloric intake, which could contribute to fight obesity. Furthermore, slow starch digestion could benefit people suffering from diabetes by slowing the postprandial blood glucose release from starch-based foods [5,6]. The non-starch polysaccharides of sorghum include insoluble fibers, mainly arabinoxylans, and soluble fibers.

Sorghum is also an important source of B group vitamins, fat-soluble vitamins like vitamin D, E, and K, and minerals [7,8]. Besides its nutritional value, sorghum has prominent potential in health-promoting. Several studies have shown the effects of sorghum and its extracts on gut microbiota, oxidative stress, cancer cell arrest, glycemic control, obesity, dyslipidemia, cardiovascular disease, and hypertension [5,9,10]. Those benefits could be due to sorghum bioactive components, such as polyphenols and bioactive lipids, especially policosanols and phytosterols [6]. Sorghum also contains anti-nutritional factors like tannins, phytic acid, trypsin inhibitor, and oxalates [11,12]. Anti-nutritional factors are not unique to sorghum but are common to all cereals. However, processing, such as germination and fermentation, can decrease anti-nutritional factors while enhancing the bioavailability of other nutrients in cereals and legumes [13,14,15].

In Africa, germination is a traditional practice and an important step for the preparation of several sorghum-based products. In Togo, as well as in other West African countries, sorghum grains are used to prepare thick porridge known under various names, such as “*koko*” in Togo, Nigeria, and Ghana, “*tò*” in Burkina Faso, or other porridges and couscous (*dambu*); while germinated sorghum is often used in the production of fermented beverages like “*tchougoudou*” in Togo and “*dolo*” in Burkina Faso, and non-fermented beverages known as “*ahoula*” are used in Togo and for infant food (thin porridge).

Germination affects the enrichment and profile of bioactive components in cereal and legume grains, which, in turn, impact the potential health effects of finished products [16,17]. Many results about the germination effects on the phenolic compounds richness have been obtained in sorghum grains. A several-fold increase in total phenolic content and antioxidant activity upon germination of sorghum has been reported in numerous works [16,18,19,20]. Conversely, a significant decrease in the total phenolic content after germination has been observed in other studies [21,22,23], whereas Dicko et al. [24] observed similar phenolic levels. Besides, gastrointestinal digestion or various food processing could favor the release of bioactive peptides that are encrypted in the native protein sequences; these peptides, once released, can exert a wide range of biological functions, such as antioxidant, anti-inflammatory, antimicrobial, antithrombotic, and hypotensive activity, e.g., inhibiting the angiotensin-converting enzyme (ACE) [25]. Recent evidence suggests the potential production of peptides with ACE-inhibitory activity after germination [17,26]. However, these changes in bioactive components and macromolecules depend on various conditions, such as soaking and germination time, and environmental conditions like humidity, temperature, light, and culture media [27,28].

This study aimed to investigate the effect of germination on bioactive compounds and the antioxidant and antihypertensive activity of sorghum from Togo. For this purpose, the total phenolic content was estimated as Folin–Ciocalteu reducing capacity, while the antioxidant activity was evaluated by 2,2-diphenyl-1-picrylhydrazyl (DPPH) and Ferric Reducing Antioxidant Power (FRAP) in vitro tests and using the cellular antioxidant activity (CAA) assay on an ex vivo system of human erythrocytes. Lastly, the antihypertensive action of sorghum peptides fraction was estimated as the ACE-inhibitory activity.

## 2. Materials and Methods

### 2.1. Chemicals and Reagents

All reagents and standards were of analytical grade. Folin–Ciocalteu reagent, gallic acid, sodium carbonate, phosphate-buffered saline (PBS), 2,4,6-Tris(2-pyridyl)-s-triazine (TPTZ), iron(III) chloride hexahydrate (FeCl_3_.6H_2_O), iron(II) sulfate heptahydrate (FeSO_4_.7H_2_O), sodium acetate, 2,2-diphenyl-1-picrylhydrazyl (DPPH), 2,2-azobis(2-amidinopropane)dihydrochloride (AAPH), dichlorofluorescein diacetate (DCFH-DA), angiotensin-converting enzyme (ACE) from rabbit lung, hippuryl-histidyl-leucine (HHL), quercetin, and acetone were purchased from Fluka-Sigma-Aldrich, Inc. (St. Louis, MO, USA), while methanol and acetic acid were bought from VWR International PBI (Milan, Italy).

### 2.2. Plant Material and Germination Process

Sorghum grains were commercially purchased at the local market of Gbonsimè in Lomé (Togo) and were a mixture of *Sorghum bicolor* varieties “Adjito”, “Kadag”, and “Minsèmè” grown locally in Togo. This product was chosen because it is mainly used in the preparation and processing of sorghum-based foods and is best-selling and most appreciated by consumers. The malting process was performed traditionally. Before germination, sorghum grains were steeped in water for 24 h in the dark. Germination was performed at room temperature for 3 days in the dark. The primary shoots and roots appearance was observed at the end of germination, and the germinated grains were dried. After that, both germinated and non-germinated sorghum grains were ground into powder using mortar and pestle. The dry flours were inspected and tested by the plant protection service of Togo, according to official protocols, which issued an import certificate attesting that materials were free from quarantine and other pests and conformed to the current phytosanitary import requirements. The inspected materials were packaged, at room temperature, shipped to Italy, and stored at 4 °C until use.

### 2.3. Plant Material Extraction

Flour samples (0.5 g) were mixed with 10 mL 70% acetone, shaken for 2 h at room temperature, and centrifuged for 10 min at 2700× *g* at 4 °C. After that, the supernatants were collected and kept at 4 °C in the dark until use. The extraction was carried out in triplicate.

### 2.4. Total Phenolic Content Determination

Total phenolic content, estimated as Folin–Ciocalteu reducing capacity, was determined in triplicate by a colorimetric method, as described by Gabriele et al. [29]. Briefly, 100 µL of germinated and non-germinated flour extracts was mixed with 500 µL of Folin–Ciocalteu reagent (diluted 1:10 with bidistilled water) and incubated for 5 min in the dark at room temperature. After that, 400 µL of sodium carbonate 0.7 M was added. After 2 h of incubation at room temperature in the dark, the absorbance was measured at 760 nm. The estimation of total phenolic content was calculated by a calibration curve obtained with gallic acid and expressed as mg of gallic acid equivalents (GAE)/g dry weight (DW).

### 2.5. Determination of Antioxidant Activity

In vitro antioxidant activity DPPH assay was carried out in triplicate, according to Colosimo et al. [30]. Briefly, 50 µL of sorghum extracts (germinated and non-germinated) was added to 1950 µL of a methanolic solution of DPPH 60 µM and mixed for 30 min at 30 °C in the dark. The absorbance was recorded at 517 nm, and the antiradical activity (ARA) was determined using the following equation: ARA = [1 − (A_S_/A_DPPH_)] × 100, where A_S_ is the absorbance of the sample, and A_DPPH_ is the absorbance of DPPH solution. The extract concentration corresponding to 50% of DPPH inhibition (EC_50_) was calculated according to Gabriele et al. [31].

FRAP assay was carried out in triplicate, as described by Colosimo et al. [30]. An aliquot of 2500 µL of freshly prepared FRAP reagent containing acetate buffer 300 mM (pH 3.6), TPTZ 10 mM in HCl 40 mM, and FeCl_3_.6H_2_O 20 mM at a ratio of 10:1:1 was added to 85 µL of extracts and gently vortexed. After 6 min of incubation at room temperature, the absorbance was measured at 593 nm, and the results were expressed as Fe^2+^ equivalents (µM) using a water solution of FeSO_4_.7H_2_O (100–2000 µM) for the calibration curve.

Ex vivo antioxidant activity. The ex vivo antioxidant activity of sorghum extracts was determined in red blood cells (RBC) using the cellular antioxidant activity (CAA-RBC) assay, as described by Frassinetti et al. [32]. Prior to the CAA-RBC test, five human blood samples from healthy patients were collected in ethylene diamine tetraacetic acid (EDTA)-treated tubes, centrifuged at 2300× *g* for 10 min at 4 °C to discard plasma, platelets, and buffy coat. Then, 2 mL of red blood cells (diluted 1:100 with PBS pH 7.4) was incubated for 1 h at 37 °C in the dark under gentle stirring with 250 µL of DCFH-DA (15 µM final concentration), and 250 µL of sorghum extracts or quercetin (8 µM) was used as standard. At the end of incubation, the RBCs were washed twice with 2 mL of PBS to remove the antioxidants remaining in the extracellular medium, resuspended in 1 mL of cold PBS, and an aliquot was transferred in a microplate. After that, AAPH solution (1.2 mM final concentration) was added to the cell suspension, and the fluorescence was analyzed at 485 nm excitation and 535 nm emission by a Victor^TM^ X3 Multilabel Plate Reader (Perkin Elmer). Values were expressed as CAA unit using the Wolfe and Liu [33] formula: CAA = 100 − (ʃSA/ʃCA) × 100, where ʃSA is the integrated area of the sample curve, and ʃCA is the integrated area of the control curve.

### 2.6. Peptide Extraction

Peptide extraction was performed in triplicate, as described by Vilcacundo et al. [34]. Flours from germinated and non-germinated sorghum were homogenized in ten-fold volume of water (1:10, *w*/*v*), and its pH was adjusted to 12 using 1 M NaOH. The homogenates were shaken for 1 h and centrifuged for 30 min at 4500× *g* at 25 °C. The supernatants were collected, and the pH was adjusted to 4.0 with 2 M HCl. Then, the suspensions were centrifuged for 20 min at 4500× *g* at 4 °C. The pellets were dissolved in water, neutralized with 0.1 M NaOH, lyophilized, and stored at −20 °C until use. The protein concentration was evaluated using the Lowry method [35].

### 2.7. Angiotensin-Converting Enzyme (ACE) Inhibitory Activity

The lyophilized peptide extracts were resuspended in 0.1 M borate buffer containing 0.3 M NaCl (pH 8.3) and analyzed for ACE-inhibitory activity, as described by Siow and Gan [36], with minor modifications. An aliquot of 50 µL of pre-diluted extracts (50 µg/mL) was added to 50 µL ACE solution (50 mU/mL) and pre-incubated for 10 min at 37 °C. Then, 150 µL of 4.15 mM substrate (hippuryl-histidyl-leucine in borate buffer) was added into the sample. After 30 min of incubation at 37 °C, the reaction was stopped by adding 500 µL of 1 M HCl, followed by the addition of 1.5 mL of ethyl acetate. The mixture was vortexed for 1 min, held 5 min at room temperature, and centrifuged. Approximately 1 mL of the ethyl acetate layer was collected and transferred into a 1.5 mL tube. The ethyl acetate was evaporated to dryness, and the residue containing hippuric acid was dissolved in 1 mL of bidistilled water; the absorbance was determined at 228 nm. Enalapril (0.4 µg/mL) and bidistilled water were used as a positive and negative control, respectively. The % ACE inhibition was calculated as follows: % ACE inhibition = (A_negative_ − A_sample_)/A_negative_ × 100, where A_negative_ is the absorbance of the negative control (bidistilled water), and A_sample_ is the absorbance of the diluted sample.

### 2.8. Statistical Analysis

Statistical analysis was performed using StatView version 5.0.1. Assays were carried out at least in triplicate, and the results were expressed as mean ± standard deviation (SD). Differences between samples were analyzed by one-way analysis of variance (ANOVA) with Fisher’s least significant difference (LSD) test. The *p*-values lower than 0.05 were considered statistically significant.

## 3. Results

### 3.1. Total Phenolic Content and In Vitro Antioxidant Activities

The total phenolic content (TPC) and the in vitro antioxidant activities of germinated and non-germinated sorghum are shown in Table 1. Regarding TPC, estimated as Folin–Ciocalteu reducing capacity and expressed as mg GAE/g dry weight, we found similar levels in the non-germinated and germinated sorghum extracts, suggesting that germination did not influence the TPC in sorghum.

Two antioxidant assays, including DPPH and FRAP, were utilized to evaluate the in vitro antioxidant activity of non-germinated and germinated sorghum. As described in Table 1, even though both non-germinated and germinated sorghum flours showed substantial antioxidant activity, non-germinated sorghum exhibited a significantly higher DPPH radical scavenging activity (*p* < 0.001), marked by a lower EC_50_ value, as well as a greater FRAP antioxidant activity (*p* < 0.0001) than the germinated one. Interestingly, although both non-germinated and germinated sorghum extracts had similar total phenolic content, they showed different antioxidant activity levels.

### 3.2. Cellular Antioxidant Activity in Red Blood Cells (CAA-RBC)

**Figure 1:**
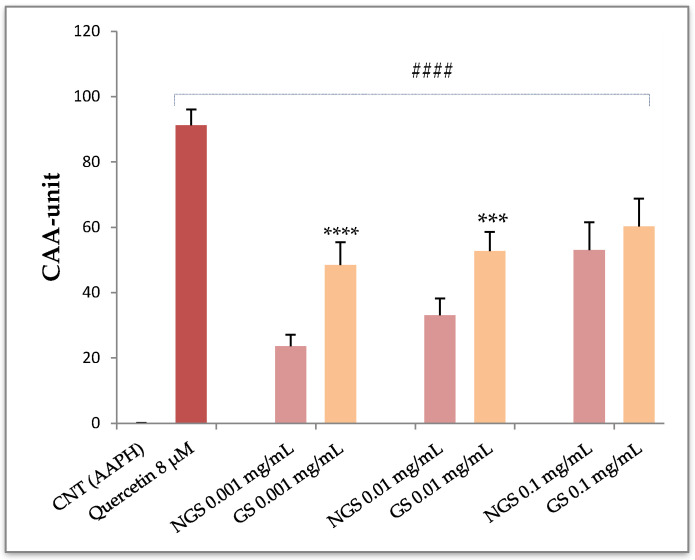
Effects of increasing doses (0.001, 0.01, and 0.1 mg/mL) of non-germinated (NGS) and germinated (GS) sorghum extracts on the cellular antioxidant activity of human red blood cells (RBCs) exposed to oxidative stress. Quercetin was used as a standard. Results were derived from five distinct healthy volunteers blood samples (*n* = 5) and expressed as mean ± SD. # Significantly different from control (CNT), #### *p* < 0.0001. * Significantly different from the co-respective NGS: *** *p* < 0.001, **** *p* < 0.0001.

In the present study, human erythrocytes were pre-treated for 1 h with three different dilutions (0.001, 0.01, and 0.1 mg/mL) of non-germinated (NGS) and germinated (GS) sorghum extracts and then exposed to a peroxyl radical generator, the AAPH. As shown in Figure 1, all treatments, as well as the quercetin used as a standard, had significantly enhanced the cellular antioxidant activity of human RBC compared to control cells that were only treated with AAPH (CAA= 0; ^####^ *p* < 0.0001). However, while a dose-dependent antioxidant effect was observed for non-germinated sorghum extracts, similar CAA values were detected following erythrocytes exposure to increasing doses of germinated ones, probably due to a saturation effect of erythrocytes antioxidant properties. As depicted in Figure 1, RBC pre-treated with GS extracts at the lowest doses exhibited a significantly higher activity than the NGS ones (48.4 ± 6.94 vs. 23.51 ± 3.53 CAA unit at 0.001 mg/mL and 52.65 ± 5.9 vs. 34.9 ± 3.23 CAA unit at 0.01 mg/mL, respectively; *** *p* < 0.001), whereas no significant differences were found at the highest tested dose (52.92 ± 8.5 NGS vs. 60.14 ± 8.6 GS CAA unit). It is interesting to note that the GS extract with the lowest antioxidant activity in the DPPH and FRAP assays exerted a generally high erythrocytes protection. Indeed, a direct comparison of the antioxidant activity of GS extract against the non-germinated one suggested that germinated sorghum could be as effective as the non-germinated in protecting RBC. Nevertheless, the germinated sorghum was more potent than non-germinated one by exerting higher antioxidant effects at lower doses than NGS, which displayed superimposable protection only at the highest dose (48.4 ± 6.94 vs. 52.92 ± 8.5 CAA unit, respectively, for GS 0.001 mg/mL and NGS 0.1 mg/mL).

### 3.3. ACE Inhibitory Activity of Sorghum Peptides

For the first time, germinated and non-germinated sorghum proteins were assessed for the ACE-inhibitory activity to determine whether germination can produce peptides with higher antihypertensive action. As expected, both non-germinated and germinated sorghum protein extracts, as well as enalapril used as a positive control, exhibited a significantly higher ACE-inhibitory activity compared to the negative control (% ACE inhibition= 0; *p* < 0.0001 for all). However, the non-germinated sorghum showed a significantly higher ACE inhibition than the germinated one (46.38 ± 2.21 vs. 15.91 ± 1.65 %, respectively; *p* < 0.0001). These results indicated that sorghum grains could be a source of ACE-inhibitory peptides.

## 4. Discussion

This study evaluated the effect of germination at room temperature for 3 days on total phenolic content, in vitro and ex vivo antioxidant activity, and antihypertensive action of *Sorghum bicolor,* grown and widely consumed in Togo. The sorghum grains herein used were a mixture of local varieties, specifically “Adjito”, “Kadag”, and “Minsèmè”, used by sorghum growers for several years. This product was chosen because it is the best-selling, most appreciated, and consumed by the local population for the preparation and processing of sorghum-based foods.

The present findings were in agreement with Dicko et al. [24], who did not detect any effect on TPC levels following sorghum germination. However, these results were in contrast with those reported by some studies. Among these, an increase in TPC after sorghum germination was described by Donkor et al. [16] and Singh et al. [20]. Conversely, Khoddami et al. [23] found a decrease in the sorghum TPC upon germination. We were not able at present to explain these apparent differences in the TPC levels since the transformation of phenolic compounds during cereal germination is not completely known. However, according to previous studies, the effect of germination on the abundance of sorghum phenolic compounds could depend on sorghum varieties (cultivar) and germination conditions, such as temperature, time of germination, humidity, etc. [19,20,24].

Besides, our results showed a decrease of in vitro antioxidant activity following sorghum germination. A similar result was also reported by Donkor et al. [16], who observed a decrement in DPPH antiradical activity after sorghum germination. Conversely, Singh et al. [20] reported an increase in DPPH inhibition activity upon sorghum germination. Several studies have shown a strong relationship between TPC and the antioxidant activity of cereal grains [16,37]. As, in our study, both non-germinated and germinated sorghum extracts had similar TPC levels, the differences in antioxidant activities could, in part, be related to the chemical composition and chemical structures of their extract components. Indeed, the antioxidant properties of an extract are not only related to its total phenolic content, but the composition and chemical structures of its components are also a major determinant of bioactivity of the extract itself [6,38].

It is well known that results from chemical assays do not always reflect the real biological effects of analyzed compounds; therefore, the antioxidant activity of both non-germinated and germinated sorghum extracts was evaluated on an ex vivo model of human red blood cells exposed to oxidative insult. The choice of erythrocytes was due to their relevant role in the antioxidant and anti-inflammatory protection of the body [39]. Moreover, RBC represents a useful cell model system since devoid of nucleus and mitochondria and, thus, the production of mitochondrial reactive oxygen species cannot influence this assay [40].

The GS extract with the lowest in vitro antioxidant activity exerted generally high erythrocyte protection. Because the exact composition of both germinated and non-germinated extracts is unknown, the greater cellular antioxidant activity of germinated sorghum could be due to some possible reasons. Since it has been reported that chemical changes in the phenolic compounds occur during seed germination [41], this process could have modified physically-chemically sorghum bioactive compounds. Therefore, these changes could, in turn, positively affect the antioxidant properties of bioactive components of the germinated sorghum extract. Secondly, studies have reported that germination generates free amino acids, short peptides due to the increase of protease activities and other components having antioxidant activity. Thirdly, the greater antioxidant activity of germinated sorghum extract may be possibly explained by a higher permeability of its bioactive compounds across the RBC membrane compared to the non-germinated ones that could be due to many factors, such as molecular weight, number and position of hydroxyl groups, and the number of double bonds [42].

Sorghum has a different composition and higher content of phenolic compounds than those observed in other cereals like wheat, barley, maize, rice, rye, and oats [43,44]. The diversity in the phenolic compound profiles correlates with the antioxidant activity and the sorghum color, which ranges from white to red, lemon yellow, and black [6,45]. Researches on sorghum have reported that its activities against oxidative stress are attributed to phenolic components and are most remarkable when black o red sorghum extracts are utilized [46,47]. The dominant class of phenolics in sorghum are phenolic acids, tannins, and flavonoids [5]. The phenolic acids of sorghum are similar to those found in other cereal grains and exhibit strong antioxidant activity, contributing to the beneficial effects in human diets [6,48]. Whereas, unlike other cereal grains, some varieties of sorghum contain condensed tannins that are mainly polymerized products of flavan-3-ols and/or flavan-3,4-diols, known as proanthocyanidins [45]. Despite their anti-nutritional effects, due to the availability reduction of sorghum minerals, proteins, and starch, which, in turn, results beneficial for diabetics, tannins are 15–30 times more effective in terms of radical scavenging capacity than simple phenolics [49]. Regarding flavonoids, sorghum is the only cereal that, besides having a high content of flavones and flavanones, contains 3-deoxyanthocyanins as the dominant compounds [6,45]. The 3-deoxyanthocyanins are more stable than other anthocyanins, common to other cereals, due to the absence of hydroxyl group at position C-3 that reduces susceptibility to nucleophilic attack and hydration [45,50]. Therefore, studies have reported that 3-deoxyanthocyanins possess unique biological properties compared to anthocyanins analogs due to their unique structure, which enhances their affinity for cellular targets relevant to disease prevention [6,51]. Sorghum contains other beneficial compounds, such as dietary fibers consisting of insoluble fibers, mainly arabinoxylans, and soluble fibers [5], which are known to influence positively the digestive tract functioning and gut microbiota. In addition, sorghum has the resistant starch, formed by the interaction between tannins and starch, which, unable to be degraded in the small intestine, reaches the large intestine acting as a soluble fiber with the potential health benefits [5,6].

Sorghum is nutritionally and functionally comparable or even superior to major cereals [5,6]. For example, as reported by Ragaee et al. [44], sorghum has shown a higher total phenolic content as well as a stronger in vitro antioxidant activity compared to hard wheat, soft wheat, barley, millet, and rye. Besides, Donkor et al. [16] reported that GS and NGS significantly inhibited α-glucosidase activity more than barley, buckwheat, oat, rye, wheat, and brown rice; whereas, among others, germinated barley and sorghum were the most potent inhibitors of α-amylase activity. In addition, GS and NGS have shown higher TPC as well as greater antioxidant capacity than oat, wheat, and brown rice.

Cereal seeds and cereal products are important sources of proteins that contain bioactive fragments, which have been reported to exert several effects, such as antihypertensive, antioxidant, anti-inflammatory, cholesterol, and lipid-lowering effects. These bioactive peptides are less active when incorporated into the parent protein sequence and could be released through three ways: enzymatic hydrolysis, digestion, and fermentation of native protein sources [25]. Therefore, enzymatic hydrolysis of proteins is the most usual way to produce bioactive peptides [26]. Kamath and colleagues [52] reported for the first time the in vitro ACE-inhibitory activity of chymotryptic hydrolysates of α-kafirin, the storage protein of sorghum, which constitutes 50–60% of total grain protein. The authors showed that four fractions of α-kafirin hydrolysates exhibited, under the experimental conditions, a significant greater ACE-inhibitory activity than those reported for hydrolysates of other cereals and legumes.

Proteolysis is an essential process for the germination of cereal seeds that involves various types of peptidases, such as endopeptidase, carboxypeptidase, aminopeptidase, etc. The activation of these enzymes results in the degradation and mobilization of storage proteins [53,54]. The proteolytic activity occurring during the germination of certain legumes has produced bioactive peptides, which exhibit a higher in vitro ACE-inhibitory activity [17,26].

It is not surprising that non-germinated sorghum proteins have shown higher ACE-inhibitory activity since various studies have reported better ACE inhibition for the crude protein isolate than digested proteins from plant seeds, suggesting the presence of pre-existing ACE inhibitory peptides in the protein extract [34,55]. Our findings are in agreement with Mamilla and Mishra [17], who showed a decrease in the ACE-inhibitory activity of red lentil germinated for 5 days at 30 °C. In contrast, Bamdad et al. [26] reported an increase in the ACE-inhibitory activity of lentil after germination at 20 °C for 5 days. However, the decreased ACE-inhibitory activity of germinated sorghum could, in part, be explained by some possible reasons. Firstly, it has been reported that the protease activity increases during the early stages of seeds germination and decreases later [19,26] that, in turn, result in a higher increase of free amino acids during 72 h of germination [19]. Hence, the decrease in ACE inhibition of germinated sorghum may be due to the breakdown of pre-existing ACE-inhibitory peptides into free amino acids, which could act as an energy source, providing carbon and nitrogen for the seed germination [41].

Secondly, endopeptidases play a crucial role in the seed germination through storage protein degradation and mobilization, producing small peptides [56] that perhaps could have biological functions. However, some ACE-inhibitory peptides released by endopeptidases could be degraded by carboxypeptidases, which can have a role during the seed germination by completing the degradation of storage proteins and degrading small peptides produced into free amino acids [56].

Finally, the decrease of ACE-inhibitory activity of sorghum upon germination may be due to environmental conditions, such as temperature and time of germination, which could affect type and proteases released at different stages of the germination process. Siow and Gan [36] reported that peptides production was time-dependent and demonstrated that the antihypertensive effect of *Parkia speciosa* enhanced with longer hydrolysis times. Besides, Aluko [57] stressed that a longer duration of hydrolysis could probably produce small size peptides with higher biological effects. Moreover, a decrease in ACE-inhibitory activity of germinated lentil was found by Mamilla and Mishra [17], while an opposite trend was observed by Bamdad et al. [26], who reported increased ACE-inhibitory activity following lentil germination under the same conditions, except for the temperatures, which were 30 °C and 20 °C, respectively. Therefore, the germination conditions used in the present study may not be suitable for obtaining sorghum products with stronger ACE-inhibitory activity.

The role of sorghum and its components in human nutrition and health has been well documented [6]. However, data on the effects of sorghum on the human health of natives in the developing countries in Africa is relatively limited. Epidemiological evidence has reported that the replacement of sorghum by maize as a staple food of the diet has raised the incidence of squamous carcinoma of the esophagus in black people of South Africa [58]. Recent human trials have reported that traditional Malian foods made from sorghum have exhibited slower gastric emptying than rice, potato, or pasta, suggesting that sorghum improves the glycemic response [59]. In addition, a study comparing gut microbiota of children in the village of rural Africa with the gut microbiota of Western European children has reported a higher microbial richness and biodiversity in rural African children than in European children, indicating that diet is partly responsible for these positive/beneficial effects [60]. Therefore, sorghum and its components may be an important contributor to this microbial richness and biodiversity since it represents the main food for children in the village of rural Africa.

## 5. Conclusions

The results obtained in this work indicate that germination of sorghum seeds had no impact on the total phenolic content, but it might have positively changed the phenolic profile, generating new bioactive compounds involved in the protection of human erythrocytes from peroxyl radicals. Thus, germinated sorghum could be, already at low concentrations, a good source of bioactive compounds effective in scavenging biological radicals underlying many chronic diseases. Furthermore, our study demonstrated the role of sorghum proteins as a source of ACE-inhibitory peptides. However, sorghum germination at room temperature for 3 days significantly reduced its antihypertensive potential. Our findings made sorghum and derived products a valuable ingredient for the formulation of novel foods with enhanced healthy attributes. Further studies will be necessary to identify suitable germination conditions for obtaining sorghum products with improved biological properties than starting material. Moreover, the analysis of the peptides profile of germinated and non-germinated sorghum will be necessary to explain changes due to the germination process as well as to individuate compounds responsible for differences in activities herein observed.

## Figures and Tables

**Table 1:** Total phenolic content and in vitro antioxidant activities of germinated and non-germinated sorghum flour. Results were expressed as mean ± standard deviation (SD) of three independent determinations.

	Total Phenolic Content (mg GAE/g DW)	DPPH EC_50_ (mg/mL)	FRAP (µM Fe^2+^)
Germinated	6.38 ± 0.37	0.51 ± 0.01	2666 ± 23
Non-germinated	6.62 ± 0.28	0.29 ± 0.05 ***	3636.2 ± 124.68 ****

Significantly different from germinated sorghum: **** *p* < 0.0001; *** *p* < 0.001. GAE: gallic acid equivalents; DPPH: 2,2-diphenyl-1-picrylhydrazyl; EC_50_: half maximal inhibitory concentration; FRAP: Ferric Reducing Antioxidant Power.

## Abbreviations

AAPH	2,2-azobis(2-amidinopropane) dihydrochloride
ACE	angiotensin-converting enzyme
CAA-RBC	cellular antioxidant activity in red blood cells
DCFH-DA	2′,7′-dichlorofluorescein diacetate
DPPH	2,2-diphenyl-1-picrylhydrazyl
FRAP	ferric reducing antioxidant power
GS	germinated sorghum
NGS	non-germinated sorghum
TPC	total phenolic content
